# Waterborne parasites: a current status from the Philippines

**DOI:** 10.1186/1756-3305-7-244

**Published:** 2014-05-28

**Authors:** Subashini Onichandran, Thulasi Kumar, Cristina C Salibay, Julieta Z Dungca, Hazel AL Tabo, Norbel Tabo, Tian-Chye Tan, Yvonne AL Lim, Nongyao Sawangjaroen, Sucheep Phiriyasamith, Hemah Andiappan, Init Ithoi, Yee-Ling Lau, Veeranoot Nissapatorn

**Affiliations:** 1 Department of Parasitology (Southeast Asia Water Team), Faculty of Medicine, University of Malaya, Kuala Lumpur, Malaysia; 2 Biological Sciences Department, College of Science and Computer Studies, De La Salle University-Dasmariñas, Dasmariñas, Philippines; 3 School of Science and Technology, Centro Escolar University, Manila, Philippines; 4 Department of Microbiology, Faculty of Science, Prince of Songkla University, Hat Yai, Thailand; 5 Graduate School, Kasem Bundit University, Bangkok, Thailand

**Keywords:** Waterborne parasites, The Philippines, Correlation, *Cryptosporidium* spp., *Giardia* spp., *Acanthamoeba* spp., *Naegleria* spp.

## Abstract

**Background:**

Despite the amount of awareness created, waterborne disease still poses threat, especially in developing countries. Due to the scarcity of reported data on waterborne parasites, the consumption of unsafe water prolongs. Thus, the occurrences of waterborne parasites from various samples were investigated from one of the Southeast Asian country, the Philippines.

**Methods:**

A total of thirty three samples, each consisting of twelve liters, were collected and processed to obtain the sediment. Ten liters of sample each was processed to detect *Cryptosporidium* spp. and *Giardia* spp. using an immunomagnetic separation method prior to enumeration via fluorescence microscope. Meanwhile, the remaining two liters were cultured to detect *Acanthamoeba* and *Naegleria* through microscopy examination and polymerase chain reaction (PCR) analysis.

**Results:**

Twelve samples (36.4%) from river (5), swimming pool (1), pond (3), rain tank (1), and natural lake (2) were positive for *Cryptosporidium* spp., 17 (45.5%) samples from river (9), pond (2), swimming pool (1), rain tank (1), and natural lake (4) were positive for *Giardia* spp. while, 13 (33.3%) samples from river (3), swimming pool (2), pond (2), dispenser (1), well (1), tap (2) and natural lake (2) were positive for *Acanthamoeba* spp. and 5 (18.2%) samples from river (1), natural lake (1), tap (1), dispenser (1) and mineral (1) were *Naegleria* spp. positive. Physical parameters such as temperature, conductivity, total dissolved solid (TDS), salinity, dissolved oxygen (DO), pH, and turbidity and chemical parameters such as ammonia, chlorine, fluoride, nitrate and nitrite were also measured. The highest chemical contamination was observed at pond 2. A good correlation was observed between *Giardia* and nitrite (r = 0.736, *p* < 0.01) and *Giardia* and nitrate (r = 0.502, *p* < 0.01).

**Conclusion:**

This study was aimed to create greater awareness of parasitic contamination in the water environment in the Philippines and also to act as a platform of the current scenario for policymakers as water pollution is a key health issue in this region.

## Background

As with many other developing countries, the Philippines is facing issues with availability of clean water and this is mainly due to factors such as, growing population, irrigation needs, rapid industrialization and urbanization, particularly in rural areas. As evidence, in 2004, 5.5% deaths were reported due to water, sanitation and hygiene-related causes. Following this, the Philippines Development Plan 2011–2016 calls for additional infrastructure investments in water, to be able to meet the growing demand [1]. However, waterborne diseases and outbreaks cannot be curbed unless sufficient level of water education is provided on the possible ways of contamination, such as through direct ingestion of contaminated water and exposure to aerosol-spray irrigation of wastewater which is often disregarded [2]. In addition, studies and reports on waterborne pathogen occurrence also need to be published in order to increase awareness of the public on this issue. Unfortunately, to date, only very few new studies had been carried out as an update to Philippines waters, particularly in detecting waterborne parasites. Thus, this study was designed to evaluate the entity of problem, if any present, and to fill in the gap of knowledge on selected water bodies in the Philippines.

## Methods

A. Collection of samples

**Figure 1:**
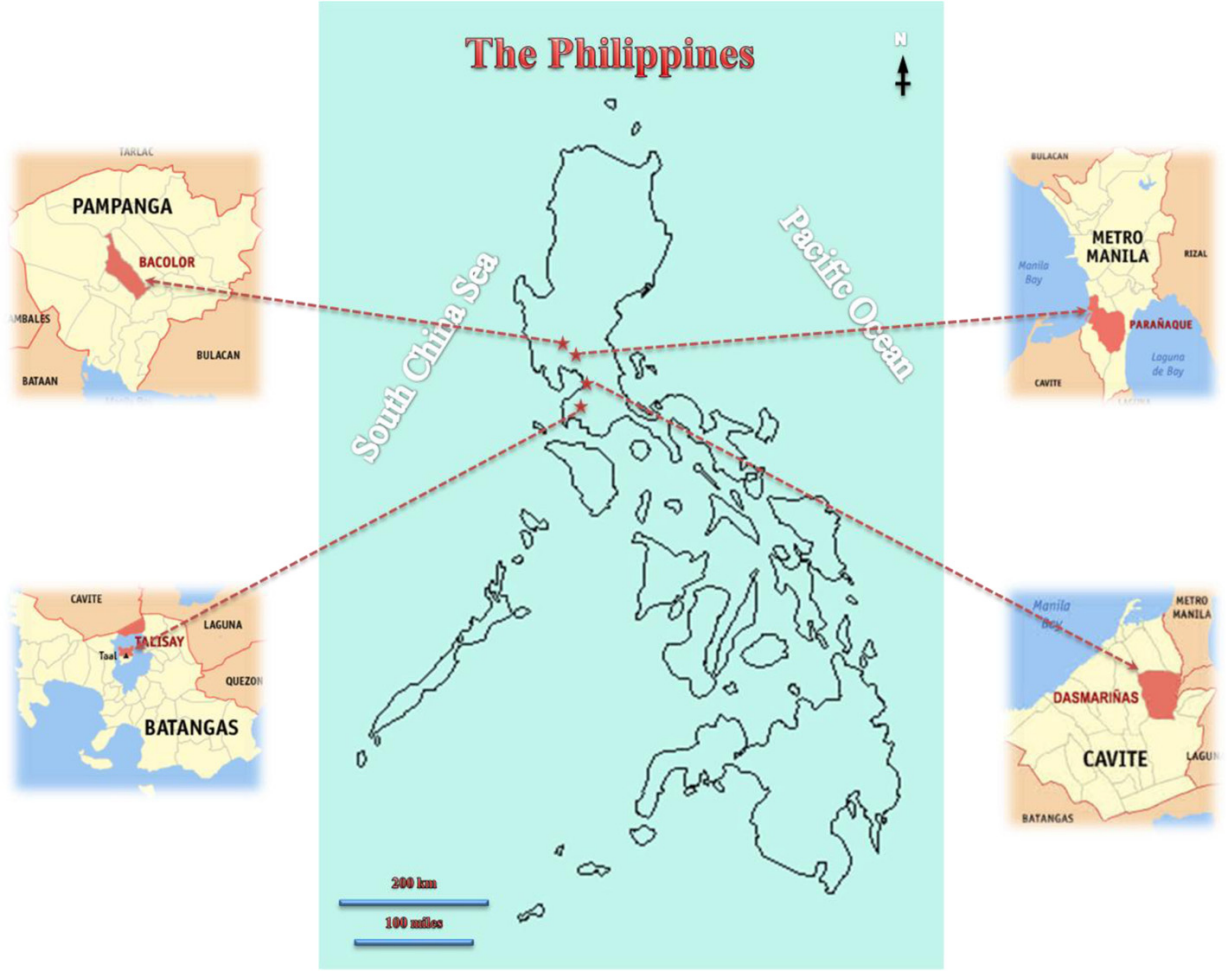
Study areas involved in the detection of waterborne parasites from the Philippines.

Overall, a total of 33 surface grab water samples were collected using 10 L and 2 L sterile polythelene bottles [3] from eleven different types of water bodies. A ten liter sample (used to screen the presence of *Cryptosporidium* and *Giardia*) and a two liter sample (used to detect the presence of *Acanthamoeba* and *Naegleria*) were collected at each sampling station. Samples were collected on October 2012 from:

a) Suburban - Batangas (Natural lake – 6 samples, well – 1 sample)

b) Suburban - Cavite (River 3&4 – 4 samples, pond 2–3 samples, swimming pool 3 – 1 sample, tap – 1 sample, drinking water – 1 sample, rain tank – 1 sample, spring – 1 sample)

c) Urban - Manila (River 2 – 3 samples, swimming pool 1&2 – 2 samples, tap – 1 sample, drinking water – 1 sample, tap storage tank – 1 sample)

d) Rural - Pampanga (River 1 – 3 samples, pond 1 – 1 sample, tap – 1 samples)Three points (upstream, midstream, downstream) were collected for rivers (Figure 1). These samples were transported in polythene bottles to the laboratory and were processed within 48 hours.

B. Measurement of physico-chemical parameters

Conductivity, dissolved oxygen (DO), pH, salinity, temperature (°C), and total dissolved solid (TDS) were measured *in-situ* at each station using a multiprobe equipment (YSI 556 MPS, USA), while turbidity was measured using a turbidity meter (ICM 2100P, USA). Chemical parameters such as ammonia, chlorine, fluoride, nitrate, and nitrite, were measured using a colorimeter (Hach DR890, USA).

C. Processing of water samples

Ten liters of water were filtered using nitrocellulose membrane (142 mm diameter, 1.2 μm pore size, Millipore, Ireland) at a flow of 250 mL/min through a flat bed membrane filtration system. Sediment trapped on the membrane filter was scraped by using an adequate amount of 0.1% Tween-80 and was aspirated to 10 mL upon centrifugation at 3000 × *g* for 15 minutes (Kubota Corporation, Japan). This was used for the screening of protozoan parasites. Separately, two liters of water sample was filtered on the same type of nitrocellulose membrane and scraped using sufficient amount of normal saline solution and was aspirated to 5 mL by centrifugation for 15 minutes at 1800 × *g*. This was subsequently used for *in vitro* cultivation of free living amoeba. All sediments obtained were stored at 4°C and were subjected to processing as soon as possible [4].

D. Measurement of rainfall

The rainfall data was obtained from http://www.samsamwater.com[5], according to locations of sampling stations.

E. Screening for *Cryptosporidium* spp. oocysts and *Giardia* spp. cysts

A commercial kit (Dynabeads GC-Combo, Invitrogen, USA) was utilized to isolate *Cryptosporidium* spp. oocysts and *Giardia* spp. cysts by using immunomagnetic separation (IMS) method. The attachment and detachment of (oo)cysts with magnetic beads was applied according to method 1623.1 [6]. The 50 μL of purified (oo)cysts were then subjected to immunofluorescence assay (IFA) through *Crypto*/*Giardia* cell, (CeLLabs, Australia). These slides were evaluated using epifluorescence microscope (Olympus BX51, Japan), whereby round, oval or ellipse shapes with bright green fluorescence (4–6 μm for *Cryptosporidium* and 8 to 18 μm × 5 to 15 μm for *Giardia*) were identified [6]. All samples that were found to be positive through FITC (Fluorescein isothiocyanate) examination by × 400 magnification are subsequently examined through a DAPI (4′,6-diamidino-2-phenylindole) filter to confirm significant characteristics (light blue/intense blue internal staining and distinct sky blue nuclei). *Crypto*/*Giardia* positive control slide (CeLLabs, Australia) was used as positive control. Negative control was prepared by using PBS (Phosphate buffered saline) solution. The presence of *Cryptosporidium* and *Giardia* were enumerated and calculated to obtain the quantity of (oo)cysts per liter, as follows:

Number of (oo)cysts per liter = Number of (oo)cysts on slide (contained by 50 μL)/10 L

F. Cultivation and identification of free living amoebae

About 1 mL (5 drops from Pasteur pipette) of the concentrated sediment from the filtered water was transferred to non-nutrient agar plates lawned with heat-killed *Escherichia coli*[7] and spread evenly, with two plates per sample bottle. Then, the plates were sealed in polythene bags and incubated under room temperature. These plates were observed daily under an inverted microscope to detect slug-like moving trophozoites with hyaline pseudopodia protrusion or double walled cysts with polygonal shaped interior or parallel inner and outer walls for 14 days. Flagellation test was carried out to detect *Naegleria* spp. [8].

G. Extraction and genotyping of free living amoebae

Cultured trophozoites and cysts are harvested [9] and subjected to DNA extraction using QIAamp DNA mini kit (Qiagen, Hilden, Germany). Amplification reactions for *Acanthamoeba* was performed using genus-specific primers which are, forward primer JDP1 (5′-GGGCCCAGATCGTTTACCGTGAA-3′) and reverse primer JDP2 (5′-ACAAGCTGCTAGGGGAGTCA-3′) [10]. As for *Naegleria*, ITS (internal transcribed spacer) gene was amplified by forward primer (5′-GAACCTGCGTAGGGATCATTT-3′) and reverse (5′-TTTCTTTTCCTCCCCTTATTA-3′) [11]. Amplified DNA was detected by 1.5% agarose gel electrophoresis and visualized under UV illumination by ethidium bromide staining.

H. Statistical analysis

Data obtained were analyzed using Statistical Package for Social Sciences version 17.0 for Windows (SPSS Inc, Chicago II, USA) software. Bivariate correlation and linear regression analysis was used to evaluate the association between the presence of waterborne parasites and physico-chemical parameters. The values of *p* < 0.01 or *p* < 0.05 were considered as statistically significant.

## Results

### Morphological examination and enumeration for *Cryptosporidium* spp. and *Giardia* spp

From the 33 samples, 12 were positive for *Cryptosporidium* spp. (36.4%) and 17 were positive for *Giardia* spp. (51.5%). Presence of both protozoan parasites was observed in river 1 (1 site), river 2 (1 site), river 3 (1 site), river 4, swimming pool 3, pond 2 (2 sites), natural lake (2 sites), and rain tank. All samples in the drinking water category were free from *Cryptosporidium* spp. and *Giardia* spp. contamination. The highest presence (0.6 oocysts/L) of *Cryptosporidium* spp. was detected in river 2 of Manila (upstream) and swimming pool 3 of Cavite, while the highest contamination (74.4 cysts/L) of *Giardia* spp. was found in river 3 of Cavite (downstream) (Table 1).

**Table 1:** Sampling site and biological characteristics of samples from assessed stations

**Sampling stations**	**Number of sample/s**	**Coordinate (Latitude, longitude)**	***Cryptosporidium *spp. (oocysts/L)**	***Giardia *spp. (cysts/L)**	**PCR results for *Acanthamoeba *spp.**	**PCR results for *Naegleria *spp.**
**Recreational water**						
** River 1**	3	15°01′35.47″N, 120°39′44.62″E	0.2	0.1	+	-
		15°01′34.03″N, 120°39′53.94″E	0.2	ND	-	-
		15°01′32.44″N, 120°40′01.62″E	ND	0.7	-	-
** River 2**	3	14°36′02.77″N, 120°59′31.28″E	0.6	58.7	-	-
		14°36′00.40″N, 120°59′28.97″E	ND	10.7	+	-
		14°36′50.63″N, 120°59′26.01″E	ND	3.5	+	-
** River 3**	3	14°19′15.18″N, 120°57′32.34″E	ND	28.6	-	-
		14°19′25.23″N, 120°57′27.89″E	ND	18.5	-	-
		14°19′36.82″N, 120°57′22.06″E	0.3	74.4	-	-
** River 4**	1	14°24′16.40″N, 120°59′50.02″E	0.5	6.4	-	+
** Swimming pool 1**	1	14°35′51.93″N, 120°59′33.92″E	ND	ND	+	-
** Swimming pool 2**	1	14°35′55.47″N, 120°59′30.72″E	ND	ND	-	-
** Swimming pool 3**	1	14°19′35.40″N, 120°57′25.04″E	0.6	0.2	+	-
** Pond 1**	1	15°01′38.95″N, 120°39′31.68″E	ND	ND	+	-
** Pond 2**	3	14°19′15.82″N, 120°57′41.31″E	0.1	0.1	+	-
		14°19′15.84″N, 120°57′41.27″E	0.2	ND	-	-
		14°19′15.67″N, 120°57′41.41″E	0.3	0.2	-	-
** Natural lake**	6	14°03′51.27″N, 120°58′37.81″E	ND	ND	-	-
		14°03′44.13″N, 120°56′31.60″E	0.4	0.1	-	-
		14°02′23.87″N, 120°57′10.12″E	ND	ND	-	-
		14°02′07.23″N, 120°00′03.91″E	0.1	0.7	+	-
		14°02′12.60″N, 120°58′34.68″E	ND	0.8	+	+
		14°03′55.36″N, 120°56′11.89″E	ND	0.2	-	+
** Rain tank**	1	14°19′15.71″N, 120°57′39.75″E	0.1	0.9	-	-
**Drinking water**						
** Tap 1**	1	15°01′37.98″N, 120°39′34.09″E	ND	ND	+	+
** Tap 2**	1	14°35′55.03″N, 120°59′31.28″E	ND	ND	+	-
** Tap 3**	1	14°19′13.46″N, 120°57′43.90″E	ND	ND	-	-
** Dispenser 1**	1	14°35′54.97″N, 120°59′30.16″E	ND	ND	-	-
** Dispenser 2**	1	14°19′13.37″N, 120°58′19.45″E	ND	ND	-	+
** Well**	1	14°02′15.53″N, 120°58′34.68″E	ND	ND	+	-
** Spring**	1	14°10′19.70″N, 120°52′09.10″E	ND	ND	-	-
** Tap tank**	1	14°35′54.54″N, 120°59′30.70″E	ND	ND	-	-
** Mineral**	1	NA	ND	ND	-	+

+ = Positive.

- = Negative.

ND = Not detected.

NA = Not Applicable.

### PCR analysis for *Acanthamoeba* spp and *Naegleria* spp

9.1% of the drinking water samples and 27.3% of the recreational water samples were positive for *Acanthamoeba* spp. while 9.1% of both drinking water and recreational water, respectively, were positive for *Naegleria* spp. Both *Acanthamoeba* spp. and *Naegleria* spp. was found in natural lake of Batangas (site 5) and tap 1 of Pampanga (Table 1). All the samples from Pampanga (river 1-site 1, pond 1 and tap 1) seemed to show the presence of *Acanthamoeba* spp.

### Physicochemical analysis

Data obtained through physical and chemical parameter analysis were compared to the regulations set by Department of Environment and Natural Resources (DENR) [12]. Water samples collected were classified according to the classification of DENR. The level of DO in all sampling stations did not meet the minimal requirement (5 mg/L). The same scenario applies to the level of ammonia (0.05 mg/L), except for dispenser 2 and tap 3. Temperature of pond 1 was out of range (31°C), pH and TDS were within range. However, fluoride was high in swimming pool 2 (1 mg/L).

### Rainfall data

The highest rainfall measurements was 292 mm (river 1 and pond 1), while the lowest measurement was 227 mm (river 2, swimming pool 1 and 2).

### Correlation analysis

The data obtained from Tables 1 and 2 were subjected to bivariate correlations and linear regression analysis. The presence of *Giardia* spp. was found to vary 54.2% in the presence of nitrite (r = 0.736, *p* < 0.01), 25.2% in the presence of nitrate (r = 0.502, *p* < 0.01), 21.8% in the presence of ammonia (r = 0.467, *p* < 0.01) and 15.6% in the presence of *Cryptosporidium* spp. (r = 0.395, *p* < 0.05).

**Table 2:** Average physico-chemical characteristics of samples from assessed stations

**Samples**	**Temperature (°C)**	**pH**	**Conductivity (μS/cm)**	**Dissolved oxygen (mg/L)**	**Salinity (ppt)**	**Total dissolved solid (mg/L)**	**Turbidity (NTU)**	**Rainfall volume (mm/month)**	**Nitrate (mg/L)**	**Nitrite (mg/L)**	**Ammonia (mg/L)**	**Chlorine (mg/L)**	**Fluoride (mg/L)**
**Recreational water**													
** River 1**	28.8	6.67	0.372	0.46^*^	0.17	0.242	10.1	292	0.05	0.057	0.56^*^	0.24	0
** River 2**	28.8	6.88	0.237	0.20^*^	0.11	0.154	13.6	227	0.97	0.197	0.73^*^	0.18	0.05
** River 3**	26.3	7.13	0.378	1.63^*^	0.18	0.245	6.9	249	0.49	0.937	0.73^*^	0.08	0.20
** River 4**	28.3	7.11	0.896	0.58^*^	0.44	0.582	10.0	239	0.06	0.086	0.73^*^	0.21	0.33
** Swimming pool 1**	28.8	6.98	0.622	0.78^*^	0.30	0.404	0.9	227	0.07	0	0.09^*^	2.20	0
** Swimming pool 2**	26.1	6.88	1.148	0.24^*^	0.57	0.746	0.5	227	0.08	0.018	0.10^*^	1.14	1.88^*^
** Swimming pool 3**	29.0	6.82	0.913	0.22^*^	0.45	0.594	0.5	249	0.55	0.012	0.08^*^	0.02	0.49
** Pond 1**	31.1^*^	6.89	0.139	0.45^*^	0.06	0.091	16.2	292	0.05	0.08	0.15^*^	0.77	0
** Pond 2**	29.0	6.86	0.334	0.12^*^	0.45	0.214	19.1	249	0.19	0.275	0.73^*^	1.25	0.64
** Natural lake**	28.9	7.33	1.380	2.19^*^	0.69	0.898	4.8	252	0.001	0.0052	0.078^*^	0.008	0.098
** Rain tank**	24.4	6.79	0.062	0.11^*^	1.40	0.040	3.1	249	0.07	0.01	0.10^*^	0.06	0.68
**Drinking water**													
** Tap 1**	27.70	6.98	0.384	0.55^*^	0.18	0.249	0.51	NA	0.01	0.001	0.52^*^	0.04	0
** Tap 2**	28.73	6.99	0.123	0.60^*^	0.06	0.080	1.80	NA	0.03	0	0.09^*^	0.31	0.14
** Tap 3**	27.80	7.15	0.345	0.57^*^	0.18	0.223	0.19	NA	0.02	0.011	0	0.27	0
** Dispenser 1**	22.91^*^	6.84	0.121	0.28^*^	0.08	0.076	2.80	NA	0.02	0.011	0.09^*^	0.46	0
** Dispenser 2**	26.50	7.28	0.341	3.04^*^	0.16	0.221	0.25	NA	0.01	0	0	0.23	0
** Spring**	26.10	7.11	0.216	0.58^*^	0.44	0.582	9.99	240	0.10	0	0.10^*^	0	0.26
** Tap tank**	26.60	7.14	0.122	1.03^*^	0.06	0.079	2.66	NA	0.10	0.011	0.10^*^	0.22	0.07
** Well**	29.70	7.30	0.635	3.18^*^	0.31	0.413	0.72	252	0.09	0.05	0.09^*^	0	0.05
** Mineral**	26.70	7.11	0.504	0.45^*^	0.24	0.328	0.08	NA	0.08	0	0.08^*^	0	0

NA = Not Applicable.

^*^ = against the DENR regulation.

## Discussion

In recent years, studies related to waterborne parasites have been published in minimal quantity and only in selected areas. Despite the study on the prevalence of protozoan parasites [13], and the finding related to drinking water from uncertain sources or drinking water sourced from a nearby dumpsite [14], waterborne outbreaks have not been reported in the Philippines [15,16]. The selection of sampling stations were generally done randomly, with the inclusion criteria of location, which covers the urban (Manila), suburban (Cavite) and rural (Batangas and Pampanga) areas. The data obtained through this study could be beneficial to estimate the prevalence of waterborne parasites in these areas, on average. The sampling was carried out during the Fall Equinox, which is also during the rainy season of the summer monsoon (May-October), whereby an increasing trend in protozoan parasites was observed [13]. The presence of free living amoeba, such as *Acanthamoeba* spp. was found to be prominent in summer [17]. The possible factors that could lead to increase in waterborne parasites during rainy seasons are contamination of drinking water and flooding of rivers and lakes with water that could be from sewage or fecal contamination.

The samples could be classified into Class AA/A/B/C (Inland) and Adopt Class AA WQG. *Cryptosporidium* spp. and *Giardia* spp. was largely detected in river 2 (upstream), which was located exactly behind areas for informal settlers and seemed not to have a proper sanitation system, with the presence of many dogs around. Meanwhile, the presence of this parasite in swimming pool 3 could be due to fecal contamination or contaminated water which could not be removed even with chlorination. The increased presence of *Giardia* spp. in river 3 (downstream) could be due to the flow of residues from nearby slaughter house. Presence of protozoan parasites from all other sampling locations could be due to contamination of fecal residues sourced from human or even animals. This could be exhibited by the location of houses which are directly discharging effluents into the nearest water sources. The number of *Giardia* spp. was usually higher than *Cryptosporidium* spp. as reported in other similar findings [18,19].

*Acanthamoeba* spp. and *Naegleria* spp. was detected in almost all recreational water stations and some drinking water samples. All the sources that indicated the presence of these amoebaes were rich in TDS, thus providing nutrition and encourage growth. Since these amoebas are heat-tolerant, they could survive adverse conditions and revive in a suitable environment. The presence of these parasites in these water locations is worrying, as it could provide a source of parasitic infection, through consumption of contaminated seafood. Site observation revealed fishing to be prominent in many rivers and lakes, and some lakes and ponds are even used as fish nurseries. Consumption of these fishes (raw or undercooked) could infect humans. Presence of pathogenic *Acanthamoeba* spp. in swimming pools could be a threat to swimmers, especially swimmers who still wears contact lenses when swimming.

Comparison of data obtained with DENR showed a difference in temperature in pond 1, which could be due to the high temperature and location in open space with limited quantity of water to cool it. The lower temperature value of dispenser 1 is because the dispensed water was readily cooled when samples were collected. All the samples were in neutral state as samples were only freshwater. This could also be seen in the low level of salinity. The low dissolved oxygen level could pose harm to aquatic organisms. Highly elevated ammonia in water could cause a decline in hemoglobin and oxygen consumption of fishes and be toxic in human, however the values recorded are not high concentrations. Possible sources of ammonia could be fertilizers, animal feed production and manufacturing of fibers, plastic and rubber. The ponds displayed high turbidity levels, and this could be due to rain wash off to the basin, as these ponds are located in areas which are cleared.

A lower amount of rainfall seemed to assist in the recovery of protozoan parasites. On the basis of bivariate analysis, a strong correlation was observed between *Giardia* spp. with nitrate and nitrite. A good correlation was also observed between *Giardia* spp. and ammonia as well as between *Giardia* spp. and *Cryptosporidium* spp. [20]. Thus, nitrate and nitrite could serve as a significant predictor for the presence of protozoan parasites as this correlation had also been found in other studies [13,21]. However, further analysis needs to be carried out with a larger sample size in order to verify this association.

Based on the result obtained, water sources in the Philippines seemed to be contaminated and needs more improvement. The presence of free living amoebas in drinking water should be given more attention in order to provide safer sources of water. Our study also revealed that the rivers, especially, with unplanned housing areas are highly contaminated water sources, which might lead to waterborne outbreaks.

Safe water is the ultimate need for the locals or even the tourists, (since Philippines attracts high number of tourists, which is about 4 million per year) thus, responsible agencies should step forward to practice and maintain regular monitoring of water quality and to ensure that policy making is strictly mandated in reviving polluted water bodies.

## Conclusion

Based on the results obtained, this study could serve as a baseline epidemiological surveillance of waterborne parasites of the Philippines. Moreover, future studies involving: a) detecting the pathogenicity of these parasites through genotyping, b) obtaining a larger sample size by collecting samples in other provinces of the Philippines and c) introducing more environmental toxicology, particularly heavy metals; should be carried out in order to provide an extensive platform for risk assessment with the occurrence of parasites contamination in the water environment. This could further help to create a greater awareness among public in general, and policymakers in particular, as water pollution is being a key health issue in the region.

## Competing interests

The authors have no financial or personal relationship with other people or organizations that could inappropriately influence or bias this paper.

## Authors’ contributions

CCS and VN designed the study. SO and TK carried out the experiment, SO contributed most on manuscript writing. CCS, JZD, VN and YALL helped in manuscript writing and editing. HA, II, NS, SP, TCT, VN and YALL provided opinions and suggestions about this manuscript while HALT and NT supplied useful information about the sampling in the Philippines. All authors read and approved the final version of the manuscript.
